# Evaluation of HA-D222G/N polymorphism using targeted NGS analysis in A(H1N1)pdm09 influenza virus in Russia in 2018–2019

**DOI:** 10.1371/journal.pone.0251019

**Published:** 2021-04-29

**Authors:** Alexey V. Danilenko, Natalia P. Kolosova, Alexander N. Shvalov, Tatyana N. Ilyicheva, Svetlana V. Svyatchenko, Alexander G. Durymanov, Julia A. Bulanovich, Natalia I. Goncharova, Ivan M. Susloparov, Vasiliy Y. Marchenko, Tatyana V. Tregubchak, Elena V. Gavrilova, Rinat A. Maksyutov, Alexander B. Ryzhikov

**Affiliations:** 1 Vector State Research Center of Virology and Biotechnology, Koltsovo, Novosibirsk Region, Russia; 2 Novosibirsk State University, Novosibirsk, Russia; Foshan University, CHINA

## Abstract

Outbreaks of influenza, which is a contagious respiratory disease, occur throughout the world annually, affecting millions of people with many fatal cases. The D222G/N mutations in the hemagglutinin (HA) gene of A(H1N1)pdm09 are associated with severe and fatal human influenza cases. These mutations lead to increased virus replication in the lower respiratory tract (LRT) and may result in life-threatening pneumonia. Targeted NGS analysis revealed the presence of mutations in major and minor variants in 57% of fatal cases, with the proportion of viral variants with mutations varying from 1% to 98% in each individual sample in the epidemic season 2018–2019 in Russia. Co-occurrence of the mutations D222G and D222N was detected in a substantial number of the studied fatal cases (41%). The D222G/N mutations were detected at a low frequency (less than 1%) in the rest of the studied samples from fatal and nonfatal cases of influenza. The presence of HA D222Y/V/A mutations was detected in a few fatal cases. The high rate of occurrence of HA D222G/N mutations in A(H1N1)pdm09 viruses, their increased ability to replicate in the LRT and their association with fatal outcomes points to the importance of monitoring the mutations in circulating A(H1N1)pdm09 viruses for the evaluation of their epidemiological significance and for the consideration of disease prevention and treatment options.

## Introduction

Influenza and other acute respiratory viral infections (ARVI) account for up to 92% of all infectious diseases [1]. There are many viruses that can cause ARVI, including the influenza viruses, respiratory syncytial virus (RSV), coronaviruses (including SARS, MERS, SARS-CoV-2), and others. These infections often affect the upper respiratory tract, but sometimes spread to the lungs, causing pneumonia, which is a life-threatening condition. Viral or viral-bacterial pneumonia is the main complication of influenza or SARS-CoV-2 infection [2, 3]. Pneumonia is a complication that often accompanies severe cases and is one of the leading causes of death in influenza cases. When pneumonia occurs, acute respiratory distress syndrome (ARDS) can develop. ARDS is considered one of the causes of the high mortality rate of highly pathogenic avian influenza and may be one of the causes of death from seasonal influenza [4].

Affinity for the corresponding receptors in the lungs allows these viruses to enter lung epithelial cells. It is known that influenza viruses bind to *α-*2,3-type sialic acid receptors (in particular, avian influenza) which are most prevalent in the lungs, or to *α-*2,6-type sialic acid receptors (in particular, seasonal influenza) which are less prevalent in the lungs [5]. In addition coronaviruses such as SARS-CoV and SARS-CoV-2 have an affinity for the АСЕ2 receptor in lungs [3].

The influenza A virus is characterized by high genetic variability, which leads to the emergence of mutants with new virological properties (such as antigenicity, virulence, drug resistance and receptor specificity) [6]. One of the most prominent amino acid positions known to be associated with receptor specificity is HA 222 A(H1N1)pdm09 (H1 numbering). Seasonal influenza viruses with D222 have a greater affinity for α-2,6-type sialic acid receptors, which are predominant in the upper respiratory tract [7]. D222G/N mutations occur at the HA receptor binding site and affect receptor specificity due to altering virus affinity to α-2,6-type and α-2,3-type sialic acid receptors. D222G/N mutations affect receptor specificity and lead to higher affinity to α-2,3-type sialic acid receptors, which are predominant in the lower respiratory tract. This feature allows viruses with these mutations to enter the lung epithelial cells and can lead to the development of pneumonia. The presence of these mutations is often associated with a fatal outcome [8, 9].

The D222G mutation has been identified in the 1918 H1N1 flu, the so-called Spanish flu. Out of the five human “Spanish” influenza infection cases with determined HA sequences three had 222D and two had 222G [10]. The D222G mutation was identified in the pre-pandemic H1N1 flu that was circulating until 2009. The A(H1N1)pdm09 “swine” flu that appeared in 2009 did not have this mutation. The D222G/N mutation that so quickly appeared in the virus may have been one of the first determinants of the virulence in the HA gene in the A(H1N1)pdm09 [11].

The A(H1N1)pdm09 virus, which appeared in 2009, caused an epidemic in the 2015–2016 season in Russia with an increased rate of severe cases of the disease, which were often fatal [12]. Increased mortality from H1N1pdm09 compared to H3N2 was observed in Russia in 2017–2019 [13, 14].

During the four epidemic seasons in Russia from 2015–2019, D222G/N mutations were observed in up to 32–57% of fatal cases [12–14], and the HA 222D/G polymorphism in A(H1N1)pdm09 was observed in 30–60% of fatal and severe influenza cases in other studies in Russia [15], Germany [8] and Italy [9, 16].

Variants of the virus with D222G/N mutations may already exist in the general pool of circulating viruses, or may appear during the process of virus replication in an infected organism.

The low occurrence of mutations in circulation and less severe cases of influenza may be explained by reduced tropism of the D222G/N variants of the virus to cells in the upper respiratory tract. It was shown that viruses with D222G/N mutations may not be detected in the upper respiratory tract in cases of severe and fatal influenza but may appear and be selected for in the lower respiratory tract. This favors the selection of mutations due to the predominance of α-2,3-type sialic acid receptors [8, 13]. The use of NGS allows the observation of minor variants and co-circulation of various mutant variants of a virus [13, 16].

The goal of this study was to investigate the presence of D222G/N mutations and other polymorphic variants in A(H1N1)pdm09 viruses by using a targeted NGS approach with a focus on the study of fatal influenza cases in Russia in the epidemic season 2018–2019. The frequency of the presence of various mutations in clinical specimens was evaluated. We observed the D222G/N mutations (proportion of virus variants with the mutations ranged from 1 to 98% in samples) only in fatal cases, with the proportion of cases with mutations being close to 60% and predominant detection of the mutations in the lower respiratory tract. Co-circulation of viruses with both D222G and D222N mutations was observed in 41% of cases with the mutations (with the frequency of each mutation being more than 1%, and ranging from 3.7 to 93.9% in individual samples). A targeted NGS approach allowed thorough analysis of the viral quasispecies present in influenza cases. The results of the study pointed to the significance of the monitoring of mutations for epidemiological analysis.

## Materials and methods

### Influenza virus isolation from nasopharyngeal swabs and autopsy material

The study of clinical material (nasopharyngeal swabs) and autopsy materials was approved by the Ethics Committee IRB 00001360 affiliated with the Federal Budgetary Research Institution State Research Center of Virology and Biotechnology “Vector” (http://www.vector.nsc.ru/eticheskiy-komitet/).

Samples were collected at the local Sanitary-and-Epidemiological Centers of the Federal Service for Surveillance of Consumer Rights Protection and Human Wellbeing (Rospotrebnadzor, https://rospotrebnadzor.ru/region/structure/str_fguz.php) after obtaining written informed consent from the patients or their close relatives in accordance with the regulations of the Russian Federation. PCR-based diagnostics of the original material for influenza virus RNA was conducted in local laboratories, and then all the positive samples were sent to the SRC VB “Vector”, Rospotrebnadzor. All samples received in the SRC VB “Vector”, Rospotrebnadzor were retested using diagnostic PCR.

Viral strains were isolated in Madin-Darby Canine Kidney (MDCK) cells by infecting a monolayer of MDCK cells [17]. Influenza A(H1N1)pdm09 viruses were isolated in the MDCK (NBL-2) parental cell line. Virus isolation was performed either from swabs and washes from the upper respiratory tract or from the lower respiratory tract autopsy material. The MDCK cell monolayer was grown in 12.5 cm^2^ cell culture flasks or in 24-well cell culture plates using the MEM cell culture growth medium containing 10% heat-inactivated fetal bovine serum, 100 μg/ml gentamicin, 2.0 mM GlutaMAX reagent and 25 mM HEPES (Thermo Fisher Scientific). Prior to specimen inoculation, the growth medium was discarded from the monolayer, and cells were washed twice with a Hanks’ Balanced Salt Solution (Thermo Fisher Scientific). Following the inoculation of the monolayer with 200 μl of a specimen, flasks or plates were placed into a CO_2_-incubator (37˚C, 5% CO_2_) for 30 minutes. After adsorption, the specimen was removed from the monolayer, and the DMEM medium (Thermo Fisher Scientific) containing 0.2% bovine serum albumin (Sigma-Aldrich), 2 μg/ml TPCK-treated trypsin (Sigma-Aldrich), 100 μg/ml gentamicin (Thermo Fisher Scientific), 25 mM HEPES (Thermo Fisher Scientific) and 2.0 mM GlutaMAX reagent (Thermo Fisher Scientific) was added. Inoculated cultures were incubated at 37˚C for 7 days. The monolayer was checked for a cytopathic effect every 24 hours. The isolates were harvested when a cytopathic effect reached its maximum spread. Following one freeze-thaw cycle, the isolate sample was used for RNA extraction and for genome sequencing. Virus isolation was considered unsuccessful if during a 7-day incubation period no signs of a cytopathic effect were observed and if the medium harvested on the 7th day after inoculation was not able to agglutinate turkey and guinea pig red blood cells.

RNA was isolated using the RIBO-sorb RNA/DNA Extraction Kit (InterLabService, Moscow, Russia). The RIBO-sorb Kit includes a set of reagents for the isolation of RNA/DNA by the method of affinity sorption on silica gel particles that is used for a wide variety of biological samples, including swabs from the respiratory tract and autopsy tissue material. First strand cDNA was prepared using the “RevertaL” Kit (InterLabService, Moscow, Russia). The enzyme used in the kit is the reverse transcriptase MMlv. First strand cDNA was used for diagnostic real-time PCR for detecting RNA of influenza A virus, which was performed using the reagent kits “AmpliSense Influenza virus A/B-FL” and “AmpliSense Influenza virus H1N1pdm2009-FL”, manufactured by the Central Research Institute of Epidemiology of the Federal Service for Surveillance of Consumer Rights Protection and Human Wellbeing (Moscow, Russia).

### Sequence analysis of influenza viruses

Sequencing was carried out at the FBRI SRC VB “Vector”, Rospotrebnadzor. To determine nucleotide sequences of viral genes and genomes, viral RNA was isolated using the RIBO-sorb RNA/DNA Extraction Kit (InterLabService, Moscow, Russia) according to the manufacturer’s instructions. Reverse transcription reactions were carried out with Uni12 primer [18] for type A viruses using the First Strand cDNA Synthesis Kit (Thermo Scientific, Lithuania) according to the manufacturer’s instructions (the enzyme used in the kit is the recombinant reverse transcriptase M-MuLV). PCR amplification of cDNA was carried out according to previously described protocols with modifications [18–20]. The BioMaster LR HS-PCR (2x) Kit (BioLabMix, Russia) was used for the amplification of cDNA. This kit contains a mixture of HS-Taq DNA polymerase and Pfu DNA polymerase for high accuracy base insertion. Detailed protocols are available upon request.

A PCR amplicon with 593 bp containing amino acid position 222 in HA (from 351 to 943 bp) of A(H1N1)pdm09 was used for targeted NGS sequencing in order to increase sequence coverage. The amplicon was sequenced either with other genome segments or by itself. Background control of MiSeq sequencing was performed using plasmid pGEM-3Zf+ and the PCR amplicon obtained from the plasmid containing 542 bp (position on the plasmid from -21 to 520 bp). Deep sequencing of amplicons was performed on an Illumina MiSeq using the MiSeq reagent kit v3 (Illumina, San Diego, US). The sequences were assembled by alignment of reads to known references with bwa-0.7.15 [21]. The obtained nucleotide sequences (major virus variant) were deposited in the Global Initiative on Sharing All Influenza Data (GISAID) database.

## Results

### In-depth study of 222D/G/N polymorphism in A(H1N1)pdm09 virus

In the 2018–2019 epidemic season an in-depth study of the 222D/G/N polymorphism in samples of the A(H1N1)pdm09 virus was carried out. For this purpose, the NGS sequencing method was optimized to increase the coverage (number of reads) of the HA region containing amino acid position 222.

To increase coverage, an additional amplicon, covering the region at position 222, was added to the samples for sequencing or was sequenced itself. Thus, the coverage increased from the usual range of 100–4,000 to more than 100,000 reads per amino acid position HA 222. Using the optimized NGS method, it was possible to quantitatively evaluate 222D/G/N polymorphism and detect other mutant variants. The detection limit of the presence of a mutation in the sample was 0.2% for coverage equal to or more than 20,000 reads per codon (which corresponded to the minimal coverage of 40 sequences per SNP). The background was estimated for each mutation using control plasmid and control amplicon. The limit of detection was determined as twice the number of the largest estimated background per mutation (which was 0.1%).

Of the 77 A(H1N1)pdm2009 cases studied (including 47 fatal cases), in 18 cases D222G/N mutations were detected in major virus variants only in lethal cases (Table 1), which accounted for 38% of all studied fatal cases. In 10 of these cases, the simultaneous presence of the D222G and D222N mutations was detected (Table 1).

**Table 1:** Analysis of the 222D/G/N polymorphism in the A(H1N1)pdm09 virus samples (D222G/N mutations in the major virus variants of the samples).

Strain	source	D	G	N	Coverage (Reads per codon)#	G and N	Pneumonia diagnosis	Risk group* (WHO)	Passage history
		222	222	222					
		%	%	%					
A/Russia/386/2019	lung	-	**98.1**	-	2252		Pneumonia (lethal)	yes	С1
A/Russia/261/2019	lung	0.2	**97.8**	-	84560		Pneumonia (lethal)	yes	С1
A/Yekaterinburg/198/2019	trachea, bronchi, lung	0.2	**97.5**	-	106325		Pneumonia (lethal)		С1
A/Vologda/332/2019	lung	0.2	**97.4**	-	94217		Pneumonia (lethal)	yes	С1
A/Russia/825/2019	trachea	3.4	**95.3**	-	21942		Pneumonia (lethal)		С1
A/Irkutsk/723/2019	trachea	0.2	**93.9**	3.7	118286	Yes	Pneumonia (lethal)		С1
A/Astrakhan/4/2019	autopsy material	1.6	**93.2**	-	1082		No data (lethal)		C1
A/Yekaterinburg/327/2019	trachea, bronchi, lung	0.3	**88.1**	9.4	120569	Yes	Pneumonia (lethal)	yes	C1
A/Vladikavkaz/35/2019	Swab	12.4	**86.6**	0.2	144221	Yes	No data (lethal)		original
A/Yekaterinburg/322/2019	trachea, bronchi, lung	2.8	**84.8**	10.1	108254	yes	Pneumonia (lethal)	yes	C1
A/Vologda/480/2019	swab	13.8	**84.1**	-	111544		Pneumonia (lethal)	yes	C1
A/Yekaterinburg/72/2019	lung	0.3	**81.6**	15.8	115634	yes	Pneumonia (lethal)		C1
A/Irkutsk/973/2019	lung	8.7	**67.6**	21.7	98692	yes	Pneumonia (lethal)	yes	C1
A/Bashkiria/1675/2019	bronchi	-	**50.7**	44.5	1857	yes	Pneumonia (lethal)		C1
A/Russia/883/2019	swab	1.9	-	**97.4**	23517		Pneumonia (lethal)	yes	C1
A/Samara/829/2019	lung	12	31.5	**54.5**	105670	yes	Pneumonia (lethal)		C1
A/Samara/483/2019	lung	17.5	29.9	**50.5**	92736	yes	Pneumonia (lethal)		C1
A/Moscow/1/2019	swab	24.2	24.1	**49.2**	66943	yes	Pneumonia (lethal)	yes	C1

The table shows the percentage of virus variants with the mutations in the sample.

(-) value is below detection limit.

*-risk group for influenza included children aged between 6 months to 5 years, older adults (aged more than 65 years), individuals with chronic medical conditions.

#-coverage is the total number of NGS sequences (reads) at amino acid position 222 in HA.

The use of the optimized method to detect the 222D/G/N polymorphism revealed the presence of these mutations at a low frequency (ranging from 1% to 47% in individual specimens) in an additional nine samples from fatal cases (Table 2).

**Table 2:** Analysis of 222D/G/N polymorphism in samples of the A(H1N1)pdm09 virus (D222G/N mutations in the minor virus variants (1–47%)).

Strain	source	D	G	N	Coverage (Reads per codon)#	G and N	Pneumonia diagnosis	Risk group* (WHO)	Passage history
		222	222	222					
		%	%	%					
A/Irkutsk/718/2019	trachea, bronchi, lung	50.5	**46.8**	0.8	116636	yes	Pneumonia (lethal)		C1
A/Khabarovsk/31/2019	lung	68.6	**29.6**	0.2	109720	yes	Pneumonia (lethal)		C1
A/Khabarovsk/1644/2018	trachea, bronchi, lung	88.3	**10.8**	0.15	144668	yes	Pneumonia (lethal)	yes	original
A/Irkutsk/1061/2019	lung	84.6	**8.7**	**5.2**	111914	yes	Pneumonia (lethal)	yes	C1
A/Irkutsk/864/2019	lung	94.3	**4.3**	-	79049		Pneumonia (lethal)	yes	C1
A/Astrakhan/2/2019	lung	48.1	**3.7**	**7.9**	127410	yes	No data (lethal)		C1
A/Yekaterinburg/116/2019	trachea	95	**3.7**	-	85721		Pneumonia (lethal)	yes	C1
А/Samara/468/2019	trachea	95.8	**2.4**	0.4	64037	yes	No data (lethal)		C1
A/Astrakhan/1/2019	lung	96.2	0.8	**1.3**	146680	yes	No data (lethal)	yes	C2

The table shows the percentage of virus variants with the mutations in the sample.

(-) value is below detection limit.

*-risk group for influenza included children aged between 6 months to 5 years, older adults (aged more than 65 years), individuals with chronic medical conditions.

#-coverage is the total number of NGS sequences (reads) at amino acid position 222 in HA.

A very high level of coverage (number of reads) of the region containing the HA 222 amino acid position and the possibility of detection of the presence of 0.2% of mutations made it possible to reveal the presence of D222G/N mutations in the minor virus variant with a frequency ranging from 0.2% to 0.6% in 14 samples from fatal cases with sufficient NGS coverage (Table 3). Samples from six fatal cases did not have sufficient coverage to detect mutations with a frequency of less than 1%.

**Table 3:** Analysis of 222D/G/N polymorphism in samples of the A(H1N1)pdm09 virus (D222G/N mutations in the minor virus variants (0.2–0.6%)).

Strain	source	D	G	N	Coverage (Reads per codon)#	G and N	Diagnosis/Outcome	Risk group* (WHO)	Passage history
		222	222	222					
		%	%	%					
A/Novosibirsk/1/2019	trachea	97.9	**0.6**	**-**	63076		Pneumonia (lethal)	yes	C1
A/Samara/527/2019	lung	88.9	**0.5**	**0.2**	109337	yes	Pneumonia (lethal)		C1
A/YANAO/1/2019	trachea	98.1	**0.4**	**0.2**	181493	yes	Pneumonia (lethal)	Yes	C1
A/YANAO/188/2019	bronchi	97.7	**0.4**	**0.2**	95414	Yes	Pneumonia (lethal)	yes	C1
A/Primorie/1/2019	lung	98.1	**0.4**	**0.2**	73654	Yes	Pneumonia (lethal)		C1
A/Novosibirsk/5/2019	trachea, bronchi, lung, spleen	97.7	**0.4**	**0.2**	107006	yes	Pneumonia (lethal)	yes	C1
A/Irkutsk/975/2019	lung	97.7	**0.3**	**0.2**	98946	Yes	Pneumonia (lethal)		C1
A/Chita/791/2019	lung	97.6	**0.3**	**0.4**	120367	yes	Pneumonia (lethal)		C1
A/Krasnodar/32/2019	bronchi	99.0	**0.4**	**-**	32970		Pneumonia (lethal)	Yes	Original
A/Yekaterinburg/17/2019	lung	98.7	**0.4**	**0.2**	132778	yes	No data (lethal)	yes	Original
A/Yekaterinburg/16/2018	lung	98.7	**0.5**	**0.2**	126670	yes	No data (lethal)		Original
A/Gatchina/43/2019	Swab	98.8	**0.4**	**0.2**	136948	yes	Pneumonia (lethal)	yes	Original
A/Yakutia/c5-29/2019	Swab	99.0	**0.3**	**-**	64446		Pneumonia (lethal)	yes	original
A/Adygea/273/2019	swab	98.8	**0.4**	**-**	127412		Pneumonia (lethal)		original

The table shows the percentage of virus variants with the mutations in the sample

(-) value is below detection limit.

*-risk group for influenza included children aged between 6 months to 5 years, older adults (aged more than 65 years), individuals with chronic medical conditions.

#-coverage is the total number of NGS sequences (reads) at amino acid position 222 in HA.

The analysis revealed a high level of HA 222D/G/N polymorphism in A(H1N1)pdm09 viruses isolated from fatal cases. Thirty-eight percent of the samples from fatal cases had D222G/N mutations in the major virus variant and, in addition, 19% of the samples from fatal cases contained these mutations in the minor virus variants, with a frequency of more than 1% (1–47%). Co-occurrence of mutations D222G and D222N (1% or more of both mutation variants in a sample) was observed in 11 samples out of 27, equivalent to 41% of the samples from fatal cases (the frequency of the mutations ranged from 4% to 97% in an individual sample). It should be noted that pneumonia was diagnosed in 81% of fatal cases (38 out of 47) and 55% of fatal cases were associated with a risk group (according to the WHO criteria) [22].

### Variation of HA 222D/G/N polymorphism in the A(H1N1)pdm09 viruses in primary material and MDCK isolates

For a more accurate assessment of the polymorphism data in the A(H1N1)pdm09 viruses, an optimized NGS analysis method was used to compare the content of D222G/N mutations in the primary material and in the A(H1N1)pdm09 virus isolates obtained using MDCK (Table 4).

**Table 4:** Variation of the HA 222D/G/N polymorphism in the A(H1N1)pdm09 viruses in primary material (pm) and isolated on MDCK.

strain	Tissue	GAT (D) original %	GAT (D) MDCK %	Ratio (original to MDCK)	GGT (G) original %	GGT (G) MDCK %	Ratio (MDCK to original)	AAT (N) original %	AAT (N) MDCK %	Ratio (MDCK to original)	Original sample, coverage#	MDCK C1, passage coverage#
A/Russia/261/2019	l	0.2	0.2	1.0	98.5	97.8	1.0	0	0	No	100130	84560
A/Vologda/332/2019	l	**76.1**	**0.2**	**380.5**	**23.1**	**97.4**	4.2	*0*.1*	0	No	50419	94217
A/Yekaterinburg/322/2019	tr,br,l	**86.9**	**2.8**	**31.0**	**11.1**	**84.8**	7.6	**1.1**	**10.1**	**9.2**	66921	108254
A/Yekaterinburg/72/2019	l	**57.8**	**0.3**	**192.7**	**22.6**	**81.6**	3.6	18	15.8	0.9	74916	115634
A/Irkutsk/973/2019	l	**65.5**	**8.7**	**7.5**	**12.5**	**67.6**	5.4	21.1	21.7	1.0	27665	98692
A/Samara/483/2019	l	**65.6**	**17.5**	**3.7**	**27.4**	**29.9**	1.1	**6**	**50.5**	**8.4**	57505	92736
A/Khabarovsk/31/2019	l	**98.1**	**68.6**	**1.4**	**0.8**	**29.6**	37.0	0.2	0.2	1.0	13840	109720
A/Moscow/1/2019	swab	**75.4**	**24.2**	**3.1**	**11.5**	**24.1**	2.1	**11.6**	**49.2**	**4.2**	99268	66943
A/Irkutsk/1061/2019	l	**98.5**	**84.6**	**1.2**	**0.6**	**8.7**	14.5	*0*.1*	**5.2**	**52.0**	28140	111914
A/Irkutsk/864/2019	l	**98.7**	**94.3**	1.0	**0.4**	**4.3**	10.8	0.2	*0*.1*	No	48521	79049
A/Astrakhan/2/2019	l	**77.3**	**48.1**	**1.6**	**0.3**	**3.7**	12.3	9.1	7.9	0.9	58798	127410
A/Astrakhan/3/2019	tr,l	**98.7**	**96.2**	1.0	**0.4**	**0.8**	2.0	**0.1**	**1.3**	**13.0**	38477	146680

The table shows the percentage of virus variants with the mutations in the sample.

*-value is below detection limit.

#-coverage is the total number of NGS sequences (reads) at amino acid position 222 in HA (l–lung, tr–trachea, br–bronchi).

Analysis of the 222D/G/N polymorphism demonstrated a difference in the frequency of amino acid substitutions between samples of primary material and MDCK-cultured isolates. There was a highly pronounced tendency for an increase in the proportion of G222 when the virus was grown from primary material in MDCK, and a tendency for an increase in the proportion of N222. These results indicate the need for sequencing for a more accurate analysis of the presence of the 222D/G/N polymorphism in the primary clinical material.

### Variation of HA 222D/G/N polymorphism in the A(H1N1)pdm09 viruses in different parts of the respiratory system (URT and LRT)

The variation in the occurrence of the HA 222D/G/N polymorphism in the A(H1N1)pdm09 viruses in different parts of the respiratory system was evaluated (Table 5).

**Table 5:** HA 222D/G/N polymorphism in the A(H1N1)pdm09 viruses in different parts of the respiratory system.

Strain (lethal cases)	original specimen	GAT (D)	GGT (G)	AAT (N)	Coverage#
		%	%	%	
A/Vologda/332/2019	swab prior to fatal outcome	98.8	0.3	-	70619
A/Vologda/332/2019	lung	76.1	**23.1**	-	50419
A/Astrakhan/2/2019	trachea	83.1	0.3	2.9	56131
A/Astrakhan/2/2019	lung	77.3	0.3	**9.1**	58798
A/Moscow/1/2019	swab prior to fatal outcome	75.4	**11.5**	**11.6**	99268
A/Vladikavkaz/35/2019	swab prior to fatal outcome	10.8	**86.6**	0.2	144221

The table shows the percentage of virus variants with the mutations in the sample.

(-) value is below detection limit.

#-coverage is the total number of NGS sequences (reads) at amino acid position 222 in HA.

The analysis revealed an increase in the frequency of the D222G mutation in the lower respiratory tract compared to the upper respiratory tract, and also that the frequency of the D222N mutation was higher in the lung compared to the trachea. D222G/N mutations were also found in swabs (nasopharyngeal and oropharyngeal) taken from patients prior to their fatal outcome.

### Detection of other mutations in position 222 of HA

NGS sequencing revealed the presence of other mutations at amino acid position 222 of HA A(H1N1)pdm09 (Table 6).

**Table 6:** Detection of other mutations in 222 position of HA of the A(H1N1)pdm09 viruses.

	Amino acid	D	N	G	Y	V	A	
strain	source	GAT	AAT	GGT	TAT	GTT	GCT	Coverage#
A/Moscow/1/2019	Swab, original material	75.4	11.6	11.5	0.05*	0.05*	**0.32**	92268
A/Astrakhan/2/2019	Lung, original material	77.3	9.1	0.3	**8.39**	**2.53**	0.01*	58798
A/Yekaterinburg/72/2019	Lung, original material	57.8	18	22.6	0.02*	0.03*	**0.80**	74916
A/Moscow/1/2019	С1	24.2	49.2	24.1	0.29	0.04*	0.96	66943
A/Astrakhan/2/2019	С1	48.1	7.9	3.7	35.6	0.82	0.02*	127410
A/Yekaterinburg/72/2019	С1	0.3	15.8	81.6	0.11*	0.03*	0.01*	115634
A/Samara/527/2019**	C1	88.9	0.5	0.2	9.5	0.05*	0.01*	109337

The table shows the percentage of virus variants with the mutations in the sample.

*-value is below detection limit.

**-Original sample data is not available.

#-coverage is the total number of NGS sequences (reads) at amino acid position 222 in HA.

The presence of other mutation variants was detected in the original material from three fatal cases. 222D/G/N/Y/V polymorphism was detected in the original sample of A/Astrakhan/2/2019 and it was also detected in the viral isolate of A/Astrakhan/2/2019 (Table 6). It was observed that the frequency of 222Y increased after cultivation of virus in MDCK. In addition, 222D/G/N/A polymorphism was detected in the original samples from two fatal cases (Table 6).

### Detection of minor A(H1N1)pdm09 virus variants with HA D222G/N mutations in samples from influenza cases with recovery

Thirty cases of influenza A(H1N1)pdm09 cases who recovered were investigated. No major variants of the virus with D222G/N mutations were detected in 30 samples. Six samples had sufficient coverage (> 20,000) to detect minor variants of D222G/N mutations (Table 7). The remaining 24 cases of A(H1N1)pdm09 who recovered had NGS coverage of 500–20000 reads, which was insufficient to detect minor virus variants with mutations.

**Table 7:** Analysis of minor A(H1N1)pdm09 virus variants with HA D222G/N mutations in samples from influenza cases with recovery.

Virus (recovery cases)	source	D	G	N	Coverage#	G and N	Diagnosis	Risk group* (WHO)	passage
		222	222	222					
		%	%	%					
A/Chelyabinsk/945/2018	swab	98.9	**0.3**	-	60371		No data (recovery)		C1
A/Kamchatka/P1/2018	swab	98.9	**0.3**	-	72586		No data (recovery)		C1
A/Samara/183/2018	swab	99.0	**0.3**	**0.2**	78744	yes	No data (recovery)		C1
A/Saratov/2231/2018	swab	99.0	**0.3**	-	52236		Pneumonia (recovery)		C1
A/Chita/1/2018	swab	99.2	**0.3**	-	49427		Pneumonia (recovery)	yes	C1
A/Adygea/254/2019	swab	98.9	**0.4**	**0.2**	162677	yes	Pneumonia (recovery)		original

The table shows the percentage of virus variants with the mutations in the sample.

(-) value is below detection limit.

*-risk group for influenza included children aged between 6 months to 5 years, older adults (aged more than 65 years), individuals with chronic medical conditions.

#-coverage is the total number of NGS sequences (reads) at amino acid position 222 in HA.

An in-depth analysis of the D222G/N mutations revealed that all six tested samples with sufficient coverage had minor variants of the virus with D222G/N mutations (0.2–0.4%).

### Analysis of the percentage of nucleotide substitutions in the A(H1N1)pdm09 genome

Analysis of the average percentage of nucleotide substitutions in the genome of the A(H1N1)pdm09 was conducted using available NGS data and control plasmid samples. For A(H1N1)pdm2009 virus samples with sequence coverage of more than 20,000 reads, the highest average percentage of substitutions was observed for the pair A to G and T to C, which amounted to 0.33% and 0.32% substitutions/nucleotide respectively, for the HA1 gene segment. The next most frequent was the pair C to T and G to A at 0.14% and 0.12%, respectively, while the percentage of mutations in the control plasmid was 0.05–0.07% for these two pairs (S1 Table). A higher percentage of the mutations A to G and T to C was observed in HA1 compared to HA2 and in all other gene segments.

## Discussion

Monitoring of A(H1N1)pdm09 viruses in Russia in the 2017–2018 epidemic season revealed the significant presence of D222G/N mutations in major and minor viral variants in samples from fatal influenza cases [13]. Since these mutations are associated with increased morbidity and mortality [8, 9, 12], an in-depth study of the polymorphism at the HA 222 amino acid position was carried out in the A(H1N1)pdm09 virus in Russia in the 2018–2019 epidemic season.

For this purpose, the NGS sequencing method was optimized in order to increase the coverage (number of reads) of the HA region containing the D222G/N mutation position (an amplicon containing the amino acid 222 codon was used for targeted NGS sequencing). Optimization of the sequencing method led to an increase in coverage by 10–50 times (up to 100,000 reads per codon) and made it possible to detect the presence of the D222G/N mutation with a detection limit of 0.2%. A similar detection limit (0.1%) was demonstrated in previous studies using Illumina for targeted sequencing [23].

Molecular genetic analysis revealed the significant presence of D222G/N mutations in the hemagglutinin receptor-binding site. The primary analysis of nucleotide sequences obtained from NGS showed the most predominant virus variant in a population of virus quasispecies in a sample. D222G/N mutations were detected in major virus variants in 38% of fatal cases (Table 1). Some researchers have suggested that the increased virulence and pathogenicity of influenza A(H1N1)pdm09 virus may be due to the presence of D222G/N mutations in HA [8, 9, 12].

Influenza virus entry into the cell occurs mainly through the binding of SA-alpha-2,3 and 2,6 cellular receptors. SA-alpha-2,6 receptors are predominantly located on the cells of the upper respiratory tract, and SA-alpha-2,3 receptors are predominantly located on the cells of the lower respiratory tract, in particular on the alveolar cells of lungs. A(H1N1)pdm09 viruses with the D222G mutation in HA have a preferential affinity for SA-alpha-2,3 receptors [11, 24, 25]. The affinity of A(H1N1)pdm09 viruses with mutation HA D222N for both SA-alpha-2,3 and SA-alpha-2,6 receptors was shown to be increased compared to the wild type, with stronger affinity for SA-alpha-2,3 receptors [24, 26]. Thus, D222G/N mutations increase virus tropism to the cells in the human lower respiratory tract, which favors the development of viral pneumonia. In animal studies, viruses with the D222G mutation have also been shown to be more virulent than the wild type [27, 28].

Analysis of the presence of polymorphism at the 222 position in the HA protein using NGS data revealed D222G/N mutations in minor virus variants (proportion of virus variants with the mutations ranged from 1% to 47%) in samples from 9 fatal cases of influenza A(H1N1)pdm09 (Table 2). Thus, D222G/N mutations (>1% in sequenced sample) were detected in 57% of the samples from all studied fatal cases. Such high levels of the D222G/N mutation in A(H1N1)pdm09 viruses from severe and fatal cases have been shown in a number of previous studies [8, 9, 15].

In addition, the use of targeted NGS revealed the low frequency of D222G/N mutations (from 0.2 to 0.6%) in samples from 14 fatal cases of influenza A(H1N1)pdm09 (Table 3).

We thus found D222G/N mutations (with a frequency of 0.2–98%) in samples from all studied fatal cases with sufficient NGS coverage (20,000 reads per codon and more) (Tables 1–3). The presence of the D222G/N mutations was also demonstrated in cases with recovery but only at a very low frequency (from 0.2 to 0.4%) (Table 7) in all samples with sufficient NGS coverage (20,000 reads per codon and more). In a previous study the presence of minor virus variants with the D222G mutation (less than 1%) was also observed in all analyzed influenza virus samples from mild cases [29]. The results of the NGS analysis indicate the importance of testing for the possible presence of D222G/N mutations in minor variants in most circulating viruses.

Analysis of the presence of major and minor virus variants with the mutations also revealed the coexistence of two mutations D222G and D222N in 41% of the studied fatal cases (mutation frequency from 1–93.9%). Previous studies have found various combinations of 222D, 222G and 222N in severe and fatal cases [8, 13, 15, 16]. The co-circulation and interaction of various virus variants of the viral population of quasispecies with the immune system can lead to rapid adaptation and escape from immune control. This may lead to a more severe course of the disease [9].

The simultaneous presence of wild-type virus and viruses with D222G/N mutations could lead to higher transmissibility of the mixture compared to a viral sample with 100% of the virus variant with D222G/N mutations, since viruses from the mixture have an affinity for both types of receptor and may enter and reproduce in both the URT and LRT.

Variants with polymorphism 222D/G/N/Y/V, 222D/G/N/A were detected in the original material from fatal cases in 2018–2019 in Russia (Table 6).

A number of previous studies have also shown the simultaneous presence of viruses with different mutations at position 222 in HA. Various combinations of 222D, G, N, Y, A, E, and V have been found in severe and fatal influenza samples [8, 15, 16, 30–33]. While D222G/N mutations are often associated with severe disease and mortality [8, 9, 12, 34], the association of D222Y/A/V/E mutations with the severity of the disease has not been established [9, 34, 35]. A meta-analysis of published data from 2009 to 2012 showed that there is a statistically significant association between the mutation D222G and severe disease and fatality. Mutation D222N was also associated with severe disease and fatality, but this association was not statistically significant and the study did not show any association of D222E with severe disease [35].

### Occurrence and frequency of mutations in A(H1N1)pdm09 genome

The frequency of mutations in the genome of the influenza A(H1N1)pdm09 virus in samples from the 2018–2019 epidemic season was analyzed. The average frequency of nucleotide substitutions in comparison with the reference sequences was calculated for the sequences of all genomic segments of the studied A(H1N1)pdm09 viruses. The most common mutations were A to G and U to C (S1 Table). The proportion of mutations in the HA1 region of hemagglutinin was higher than in the HA2 region and other genomic segments, but the difference was not statistically significant. It is known that mutations accumulate faster in HA1 than in HA2 [36]. The pair of mutations observed from A to G and U to C likely arise due to A–C mispairing during RNA replication. During the synthesis of negative-strand RNA from positive-strand RNA, A mispairs with C. The positive-strand RNA, which is synthesized from the mutated negative-strand RNA, thus contains the G nucleotide. Similarly, for mutation U to C to occur in positive-strand RNA, first the negative-strand RNA is synthesized with nucleotide A and after that A to C mispairing occurs during synthesis of the positive-strand RNA, which results in mutation U to C in positive-strand RNA.

The D222G mutation occurs in the hemagglutinin (HA) gene in the HA1 region. The D222G mutation is caused by an A to G transition at the second codon position (GAT to GGT), and the mutation D222N results from a reverse G to A transition at the first codon position (GAT to AAT). According to the analysis of nucleotide mutation frequency, mutation A to G occurs more often than G to A (S1 Table). According to another study, the transition from G to A occurs six times less often than A to G [37]. This may contribute to the higher occurrence of the D222G mutation compared to D222N and other variants.

In the studied samples, mutation D222G was observed more often than D222N in samples from fatal cases (14 D222G and 4 D222N in major virus variant) (Table 1). Also, the D222G mutation was more common than D222N in samples from fatal cases in other studies. Moreover, the D222N mutation was often present in the minor variant [8, 15].

An increase in the frequency of D222G/N mutations was found in the lungs compared to the upper parts of the respiratory system (Table 5). Similar observations have been made in a number of previous studies [16, 32]. The results of these studies support the hypothesis that mutations arise as a result of selection from polymorphic influenza viruses in the lower respiratory tract. At the stage of infection, D222G/N mutations may be absent or not dominant. Differences in virus populations in different body tissues have also been shown for other viruses [38].

In the analysis of the samples from the 2018–2019 epidemic season, a major virus variant with mutation D222G (87%) was detected in one swab from a fatal case taken prior to fatal outcome (Table 5); in addition, minor virus variants with mutations D222G (11.5%) and D222N (11.6%) were detected in one swab from a fatal case taken prior to fatal outcome (Table 5). These types of cases are rare and it is likely that the mutated virus appeared in the URT by moving from the LRT, where the selection of the mutations had occurred. The presence of a significant proportion of viruses with D222G/N mutations in the URT may lead to the adaptation and evolution of the viruses to the URT receptors while preserving affinity to the LRT receptors. This could lead to the increased transmissibility of potentially more pathogenic virus variants. This observation highlights the importance of monitoring the circulation of viruses with these mutations.

### Epidemiological significance of D222G/N mutations

An analysis of influenza morbidity in Russia in the 2018–2019 season revealed that among fatal cases A(H1N1)pdm09-associated illness was more prevalent (70%) than A(H3N2) infection, while in the general circulation there was an approximately equal distribution of influenza A(H1N1)pdm09 and A(H3N2) among type A viruses [14]. A similar increased proportion of A(H1N1)pdm09-associated fatal cases compared to the proportion of A(H1N1)pdm09 in general circulation was reported for the 2017–2018 epidemic season [13]. A similar higher proportion of A(H1N1)pdm09 among intensive care unit (ICU) cases was observed in a study in Italy in the 2014–2015 epidemic season [16]. Due to the increased mortality associated with А(H1N1)pdm09 observed in some reports it is important to study pathogenicity markers such as HA D222G/N and other virulence and pathogenicity markers [8, 13, 16, 35]. The presence of D222G/N was discovered in a significant proportion of А(H1N1)pdm09 fatal cases. At the same time, there were many fatal cases without these mutations, in which other determinants of virus pathogenicity or the patient’s risk group may have played a role. More research is needed to understand the role of D222G/N mutations in A(H1N1)pdm09 pathogenicity. The importance of epidemiological monitoring of D222G/N mutations was recognised by the WHO in 2009 [39].

The D222G/N mutations were not detected in non-lethal cases in major or minor virus variants at frequencies of more than 1% in a sample in influenza cases in Russia during the 2017–2019 epidemiological seasons [13, 14]. Other studies also note the identification of these mutations mainly in severe and fatal cases. According to monitoring no widespread circulation of these mutations has been observed [8, 9, 40]. This observation is supported by the analysis of A(H1N1)pdm09 HA gene sequences deposited globally in the GISAID database during the 2009–2019 epidemic seasons.

Despite the low prevalence of viruses with D222G/N mutations in the general circulation, it is important to monitor the circulation of viruses with these mutations for epidemiological prognosis. The mutations can either arise spontaneously after infection of the human host, or they can be selected for if they are present at the time of infection. If influenza viruses with D222G/N mutations are already present during an infection, then the accumulation of viruses with the mutations can occur more rapidly, contributing to disease severity and the development of clinical sequelae. There is limited information available about the estimated time of mutation accumulation in influenza cases [8]. It can be assumed that if viruses with the mutations start to broadly circulate in epidemiologically significant proportions, then the general influenza virus population may become more dangerous, especially for the risk group. Thus, monitoring influenza viruses A(H1N1)pdm09 for the presence of D222G/N mutations in circulation, including assessing the proportion of minor virus variants, would be of epidemiological significance.

In addition, the observed accumulation of viruses with D222G/N mutations in the lungs of patients with severe influenza can lead to the release of the mutated virus from the lungs into the upper respiratory tract and this may possibly lead to greater danger in persons in contact with those with the disease, especially those in the influenza risk group.

It should be noted that the risk group for severe disease caused by influenza A(H1N1)pdm09 viruses with 222D/G/N polymorphism should include people with underlying lung conditions associated with an increased number of regenerating type 2 pneumocytes, which provide preferential receptor binding for viruses with D222G/N mutations (e.g. COPD and other lung damaging conditions) [11, 41].

The development of effective diagnostics is important for monitoring these mutations during epidemic seasons [13, 42]. The study of D222G/N mutations is important for assessing their effect on the virological properties of the A(H1N1)pdm09 virus.

Vaccination is an effective form of influenza control. A relatively high vaccine effectiveness was noted for the A(H1N1)pdm2009 vaccine component in the 2018–2019 epidemiological season [14, 43]. The absence of a significant effect of D222G/N mutations on the antigenic properties of the virus was shown in our previous studies [13, 14] and another study [42]. This indicates that the vaccine remains effective in protecting against variants of viruses with D222G/N mutations.

## Conclusions

The application of targeted NGS sequencing enabled us to conduct a thorough evaluation of А(H1N1)pdm09 HA D222G/N mutations, which correlate with high morbidity and mortality. This study revealed a high proportion (38%) of cases with major virus variants with these mutations among patients with a lethal outcome. In addition this analysis revealed the presence of the D222G/N mutations at a frequency of 0.2–98% in samples from all studied fatal cases that had sufficient NGS coverage. These mutations were also found in cases with recovery at very low frequencies of 0.2 to 0.4%. Analysis of nucleotide substitutions showed that on average the substitution A-G is more predominant than other substitutions. This may lead to an overall higher probability of the origin of the mutation D222G (GAT-GGT) in contrast to D222N (GAT-AAT). The ubiquitous presence of the D222G/N mutations revealed in all studied samples (fatal and non-fatal) may indicate that they either come from a limited circulation of the viruses with the mutations in the human population or that they can arise and be further selected for in the host’s lower respiratory tract. This may occur due to the high mutation rate in the influenza A virus genome and due to the high prevalence of α2,3-linked sialic acid receptors, specific for D222G/N-hemagglutinin binding, in the lower respiratory tract.

Continuous monitoring of HA 222D/G/N polymorphism in A(H1N1)pdm09, including the evaluation of the presence of minor virus variants with mutations, is important for understanding virus evolution and the detection of an increase of viruses with the mutations in broad circulation. This data should be available for epidemiological analysis and prognosis.

## Supporting information

S1 Table Average mutation frequency per nucleotide position for gene segments of A(H1N1)pdm09 (with the division of hemagglutinin into HA1 and HA2). (DOCX)
