# Supplementary material for: Evaluation of HA-D222G/N polymorphism using targeted NGS analysis in A(H1N1)pdm09 influenza virus in Russia in 2018–2019
Source: PLoS One. 2021 Apr 29;16(4):e0251019. doi: 10.1371/journal.pone.0251019 (PMC8084186; doi:10.1371/journal.pone.0251019)
Supplement: S1 Table — (DOCX) [file pone.0251019.s001.docx]

S1 Table. Average mutation frequency per nucleotide position for gene segments of A(H1N1)pdm09 (with the division of hemagglutinin into HA1 and HA2).

| gene | H1 HA1 | SD | H1 HA 2 | SD | H1 NA | SD | H1 PB2 | SD | H1 PB1 | SD | H1 PA | SD | H1 NP | SD | H1 NS | SD | H1 M | SD | control | SD |
| --- | --- | --- | --- | --- | --- | --- | --- | --- | --- | --- | --- | --- | --- | --- | --- | --- | --- | --- | --- | --- |
| A-G | 0,33 | 0,08 | 0,25 | 0,08 | 0,25 | 0,1 | 0,23 | 0,05 | 0,24 | 0,09 | 0,26 | 0,08 | 0,3 | 0,06 | 0,27 | 0,1 | 0,2 | 0,02 | 0,070 | 0,04 |
| T-C | 0,32 | 0,09 | 0,23 | 0,09 | 0,25 | 0,09 | 0,21 | 0,06 | 0,25 | 0,09 | 0,25 | 0,08 | 0,24 | 0,05 | 0,24 | 0,09 | 0,22 | 0,02 | 0,070 | 0,04 |
| C-T | 0,12 | 0,02 | 0,11 | 0,02 | 0,11 | 0,04 | 0,1 | 0,02 | 0,11 | 0,02 | 0,11 | 0,02 | 0,1 | 0,02 | 0,11 | 0,03 | 0,1 | 0,01 | 0,050 | 0,02 |
| G-A | 0,14 | 0,04 | 0,11 | 0,04 | 0,12 | 0,05 | 0,12 | 0,02 | 0,13 | 0,04 | 0,12 | 0,04 | 0,11 | 0,02 | 0,13 | 0,05 | 0,1 | 0,02 | 0,060 | 0,04 |
| A-C | 0,05 | 0,02 | 0,04 | 0,02 | 0,04 | 0,02 | 0,04 | 0,02 | 0,04 | 0,02 | 0,05 | 0,02 | 0,04 | 0,02 | 0,04 | 0,02 | 0,04 | 0,01 | 0,050 | 0,01 |
| T-G | 0,05 | 0,02 | 0,06 | 0,03 | 0,06 | 0,03 | 0,07 | 0,02 | 0,06 | 0,02 | 0,06 | 0,02 | 0,05 | 0,02 | 0,06 | 0,03 | 0,05 | 0,02 | 0,050 | 0,01 |
| C-A | 0,08 | 0,05 | 0,09 | 0,04 | 0,09 | 0,06 | 0,07 | 0,02 | 0,07 | 0,04 | 0,08 | 0,04 | 0,08 | 0,03 | 0,09 | 0,05 | 0,07 | 0,02 | 0,060 | 0,02 |
| G-T | 0,05 | 0,02 | 0,06 | 0,02 | 0,06 | 0,02 | 0,08 | 0,02 | 0,06 | 0,02 | 0,06 | 0,02 | 0,05 | 0,02 | 0,06 | 0,03 | 0,05 | 0,01 | 0,050 | 0,02 |
| C-G | 0,03 | 0,02 | 0,03 | 0,02 | 0,03 | 0,03 | 0,02 | 0,01 | 0,02 | 0,01 | 0,03 | 0,02 | 0,03 | 0,02 | 0,03 | 0,03 | 0,03 | 0,01 | 0,030 | 0,012 |
| G-C | 0,02 | 0,01 | 0,02 | 0,01 | 0,02 | 0,02 | 0,02 | 0,01 | 0,02 | 0,01 | 0,03 | 0,02 | 0,02 | 0,01 | 0,03 | 0,02 | 0,02 | 0,01 | 0,030 | 0,02 |
| T-A | 0,07 | 0,03 | 0,06 | 0,03 | 0,07 | 0,03 | 0,06 | 0,02 | 0,06 | 0,03 | 0,06 | 0,03 | 0,06 | 0,02 | 0,07 | 0,04 | 0,05 | 0,01 | 0,040 | 0,01 |
| A-T | 0,05 | 0,01 | 0,05 | 0,01 | 0,05 | 0,02 | 0,04 | 0,01 | 0,05 | 0,01 | 0,05 | 0,01 | 0,04 | 0,01 | 0,05 | 0,02 | 0,04 | 0,01 | 0,030 | 0,003 |
